# The Clarithromycin Susceptibility Genotype Affects the Treatment Outcome of Patients with Mycobacterium abscessus Lung Disease

**DOI:** 10.1128/AAC.02360-17

**Published:** 2018-04-26

**Authors:** Qi Guo, Haiqing Chu, Meiping Ye, Zhemin Zhang, Bing Li, Shiyi Yang, Wei Ma, Fangyou Yu

**Affiliations:** a Tongji University School of Medicine, Shanghai, China; b Department of Respiratory Medicine, Shanghai Pulmonary Hospital, Tongji University School of Medicine, Shanghai, China; c State Key Laboratory of Microbial Metabolism, and School of Life Sciences and Biotechnology, Shanghai Jiao Tong University, Shanghai, China; d Department of Clinical Laboratory Medicine, Shanghai Pulmonary Hospital, Tongji University School of Medicine, Shanghai, China; e Shanghai Key Laboratory of Tuberculosis, Shanghai Pulmonary Hospital, Tongji University School of Medicine, Shanghai, China

**Keywords:** Mycobacterium abscessus, clarithromycin, genotype, lung disease, treatment outcome

## Abstract

Mycobacterium abscessus accounts for a large proportion of lung disease cases caused by rapidly growing mycobacteria. The association between clarithromycin sensitivity and treatment outcome is clear. However, M. abscessus culture and antibiotic susceptibility testing are time-consuming. Clarithromycin susceptibility genotyping offers an alternate, rapid approach to predicting the efficacy of clarithromycin-based antibiotic therapy. M. abscessus lung disease patients were divided into two groups based upon the clarithromycin susceptibility genotype of the organism isolated. A retrospective analysis was conducted to compare the clinical features, microbiological characteristics, and treatment outcomes of the two groups. Several other potential predictors of the response to treatment were also assessed. Sixty-nine patients were enrolled in the clarithromycin-resistant genotype group, which included 5 infected with *rrl* 2058-2059 mutants and 64 infected with *erm*(41)T28-type M. abscessus; 31 were in the clarithromycin-sensitive group, i.e., 6 and 25 patients infected with genotypes *erm*(41)C28 and *erm*(41) M type, respectively. The results showed that lung disease patients infected with clarithromycin-sensitive and -resistant M. abscessus genotypes differed significantly in clarithromycin-based combination treatment outcomes. Patients infected with the clarithromycin-sensitive genotype exhibited higher initial and final sputum-negative conversion and radiological improvement rates and better therapeutic outcomes. Multivariate analysis demonstrated that genotyping was a reliable and, more importantly, rapid means of predicting the efficacy of clarithromycin-based antibiotic treatment for M. abscessus lung disease.

## INTRODUCTION

The incidence of infections by nontuberculosis mycobacteria (NTM) has increased significantly in recent years (1–3). Of all NTM infections, treatment of Mycobacterium abscessus infections is the most challenging (4, 5). M. abscessus accounts for 65 to 80% of the cases of lung disease caused by rapidly growing mycobacteria and has emerged as an important pathogen for patients with bronchiectasis, chronic obstructive pulmonary disease, and cystic fibrosis (6–11).

M. abscessus is among the most antibiotic-resistant pathogens known (12). Although some antibiotics, such as amikacin, cefoxitin, and imipenem, are effective, only clarithromycin (CLA) exhibits convincing evidence of clinical efficacy for treatment of M. abscessus lung disease (8). Currently, CLA is the only effective antibiotic administered orally and, therefore, recommended as the core agent for treatment of M. abscessus infections (8).

Genotypic variations influence the sensitivity of M. abscessus to CLA. Two genotypes confer CLA resistance: a point mutation (A to C or A to G) in the 2058-2059 locus of the 23S rRNA (*rrl*) gene confers acquired resistance (13). An intact *erm*(41) gene, which exhibits a T/C polymorphism at the 28th nucleotide, confers inducible resistance when the 28th nucleotide is thymidine [*erm*(41)T28] (14, 15). Alternatively, CLA sensitivity is conferred when cytidine is the 28th nucleotide in intact *erm*(41), i.e., genotype *erm*(41)C28 (15). Deletion of *erm*(41) nucleotides 64 and 65, or deletion of nucleotides 159 to 432, also results in the loss of *erm*(41) gene function (M type) and a gain in CLA sensitivity (14, 16).

M. abscessus can be divided into M. abscessus subsp. abscessus and M. abscessus subsp. massiliense based upon the integrity or absence of the *erm*(41) gene. Korean and Japanese researchers first reported that M. abscessus subsp. abscessus and M. abscessus subsp. massiliense exhibited disparate clinical and microbiological characteristics (17, 18). Retrospective analysis and a prospective study conducted in 2017 confirmed these results and suggested that patients infected with a CLA-sensitive [*erm*(41)C28] genotype had a prognostic advantage (19, 20). Therefore, differences in the A and M subtypes may be due largely to genotypic differences that affect CLA sensitivity (21–23). Here, we report the results of a retrospective analysis undertaken to determine the relationship between genotype, CLA sensitivity, and the outcome of CLA-based treatment of M. abscessus lung disease.

## RESULTS

### Patient characteristics.

One hundred M. abscessus lung disease patients who conformed to our recruitment criteria were enrolled and divided into CLA-resistant and -sensitive genotype groups according to the *rrl* and *erm*(41) sequevar. Sixty-nine (69%) patients were enrolled in the CLA-resistant genotype group, which included 5 (7.2%) *rrl* 2058-2059 mutant- and 64 (92.8%) *erm*(41)T28-type-infected patients; 31 (31%) belonged to the CLA-sensitive genotype group, which included 6 (19.4%) *erm*(41)C28- and 25 (80.6%) *erm*(41) M-type-infected patients. No significant differences were found in the ages and genders of the two groups (Table 1). The proportion of patients with hemoptysis was higher in the CLA-resistant genotype group than the CLA-sensitive genotype group (22/69 versus 4/31; *P* = 0.045). Furthermore, a significantly greater incidence of cavity-like manifestations occurred in computed tomography (CT) scans of patients infected with isolates with the CLA-resistant genotype than in patients infected with isolates with the CLA-sensitive genotype (50/69 versus 8/31; *P* < 0.001). CT scans of the CLA-sensitive-group patients, on the other hand, displayed a higher incidence of a tree-in-bud pattern (14/31 versus 16/69; *P* = 0.027).

**TABLE 1:** Baseline characteristics of patients infected with M. abscessus belonging to CLA-resistant and -sensitive genotypes

Characteristic^*a*^	Value^*b*^		*P* value
	CLA-resistant group (*n* = 69)	CLA-sensitive group (*n* = 31)	
Median age (IQR) (yr)	58 (44–66)	56 (32–64)	0.562
Males	26 (37.7)	17 (54.8)	0.107
BMI (mean ± SD) (kg/m^2^)	19.93 ± 0.37	19.69 ± 0.57	0.729
Underlying disease			
Prior tuberculosis^*c*^	29 (42.0)	16 (51.6)	0.373
COPD	1 (1.4)	2 (6.5)	0.226
Hypertension	11 (15.9)	3 (9.7)	0.601
Diabetes	2 (2.9)	4 (12.9)	0.135
CHD	4 (5.8)	0 (0)	0.414
Malignancy	3 (4.3)	0 (0)	0.550
History of surgery	3 (4.3)	1 (3.2)	1
Symptoms			
Cough	55 (79.7)	25 (80.6)	0.914
Sputum	69 (100.0)	31 (100.0)	1
Fever	15 (21.7)	4 (12.9)	0.298
Hemoptysis	22 (31.9)	4 (12.9)	0.045
Radiographic features			
Extent			0.404
Bilateral involvement	58 (84.0)	28 (90.3)	
Unilateral involvement	11 (15.9)	3 (9.7)	
Median no. of lobes (IQR)	4 (3–6)	4 (2–6)	0.419
Disease pattern			
Bronchiectasis	66 (95.7)	29 (93.4)	0.655
Cavity	50 (72.5)	8 (25.8)	<0.001
Nodules (diam < 1 cm)	38 (55.0)	19 (61.3)	0.561
Nodules (diam > 1 cm)	39 (56.5)	16 (51.6)	0.648
Tree-in-bud pattern	16 (23.2)	14 (45.2)	0.027
Initial AFB smear positivity	28 (40.6)	10 (32.3)	0.428
Initial morphotype			0.770
Rough	40 (58.0)	17 (54.8)	
Smooth	29 (42.0)	14 (45.2)	

a COPD, chronic obstructive pulmonary disease; CHD, coronary heart disease; AFB, acid-fast bacilli; IQR, interquartile range.

b Data are the numbers (%) of patients found in the CLA-resistant and -sensitive genotype groups unless otherwise indicated.

c Patients treated for tuberculosis prior to the diagnosis of M. abscessus lung disease.

### Colony morphology.

M. abscessus isolates manifest two distinct colony morphotypes: smooth and rough. The colony morphology of the isolates associated with both CLA susceptibility groups did not differ significantly (Table 1). Patients infected with M. abscessus characterized by a rough-type colony exhibited a higher incidence of cavities in CT images (*P* = 0.043) (Table 2). The colony morphotype did not exert a significant effect on any of the other parameters assessed.

**TABLE 2:** Relationship between morphotype, results of initial CT scan, and treatment outcome

Parameter	No. (%) of patients^*a*^		*P* value
	Rough *n* = 57	Smooth *n* = 43	
Radiographic features			
Bronchiectasis	56 (98.2)	39 (90.7)	0.211
Tree-in-bud pattern	18 (31.6)	12 (27.9)	0.692
Cavity	38 (66.7)	20 (46.5)	0.043
Radiological improvement	26 (45.6)	21 (48.8)	0.749
Sputum conversion to negativity	21 (36.8)	19 (44.2)	0.458
Treatment effectiveness	31 (54.4)	25 (58.1)	0.708

a Number (percentage) of patients infected with isolates that give rise to rough and smooth colony types versus the disease parameter listed.

### Comparison of antibiotic sensitivity.

The sensitivity of all the M. abscessus isolates to 10 antibiotics tested is shown in Table 3 and Table S1 in the supplemental material. The five *rrl* 2058-2059 mutant isolates exhibited acquired resistance to CLA, i.e., they were resistant on day 3 of exposure and prior to induction. Twenty-seven of the 64 *erm*(41)T28 isolates also exhibited acquired resistance; 36 isolates were induced by 14 days exposure to CLA; and one isolate showed abnormal CLA sensitivity despite expressing an *erm*(41)T28 gene, albeit with a wild-type *rrl* gene. In sharp contrast, no CLA resistance was observed within the CLA-sensitive genotype group. Notably, although most isolates in the CLA-resistant genotype group were insensitive to CLA, only one isolate was insensitive to amikacin treatment. A considerable number of isolates in both the CLA-sensitive and CLA-resistant genotype groups were sensitive to linezolid. A large number of isolates in both groups were resistant to moxifloxacin, doxycycline, imipenem, and tobramycin; no significant difference in resistance to these antibiotics was found between groups.

**TABLE 3:** Antibiotic resistance of all M. abscessus isolates^*a*^

Isolate group (*n*)	Antibiotic	No. of isolates/MIC (mg/ml) of:												No. (%) resistant isolates^*b*^
		0.06	0.125	0.25	0.5	1	2	4	8	16	32	64	128	
Resistant (69)	Clarithromycin before induction				7	11	13	10	9	19				28 (40.6)
	Clarithromycin after induction							1		68				68 (98.6)
	Amikacin						2	13	35	10	8	1		1 (1.4)
	Linezolid					1	3	2	10	20	33			33 (47.8)
	Moxifloxacin						1	3	65					68 (98.6)
	Doxycycline							1		68				68 (98.6)
	Imipenem									3	14	52		66 (95.7)
	Tobramycin							9	22	38				60 (87.0)
	Cefoxitin										3	23	43	43 (62.3)
	Sulfonamides				2	7	22	22	16					38 (55.1)
	Tigecycline			4	10	30	16	9						ND
Sensitive (31)	Clarithromycin before induction	2	9	4	8	3			3	3				6 (19.4)
	Clarithromycin after induction		7	4	7	4	1		4	4				8 (25.8)
	Amikacin						2	3	15	9	1	1		1 (3.2)
	Linezolid							1	7	10	13			13 (42.0)
	Moxifloxacin			1				2	28					30 (96.8)
	Doxycycline							1		30				31 (100)
	Imipenem									1	4	26		30 (96.8)
	Tobramycin						1		9	21				30 (96.8)
	Cefoxitin										2	7	22	22 (71.0)
	Sulfonamides				1	5	7	10	8					18 (58.1)
	Tigecycline			2	6	10	7	6						ND

a The *erm*(41) sequevar-dependent resistance of 100 M. abscessus isolates to the antibiotics indicated was determined by the microdilution method. The incubation time was 3 days (before) and 14 days (after) induction for CLA and 3 days for the other antibiotics listed.

b Resistant isolates were distinguished according to the breakpoint provided by NCCLS document M24-A2. ND, no data. Tigecycline has no recommended breakpoint.

### Combination antibiotic treatment and treatment response.

**FIG 1:**
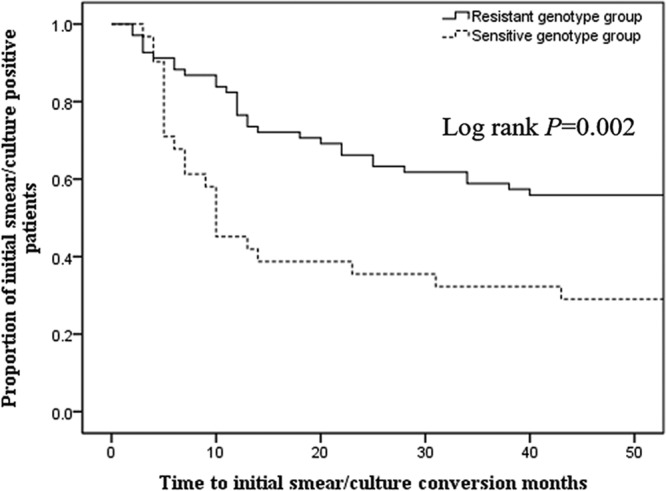
Comparison of initial sputum smear/culture conversion between patients infected with the CLA-resistant [2058-2059 *rrl* mutant or *rrl* wild type/*erm*(41)T28] and -sensitive [*rrl* wild type/*erm*(41)C28 or *rrl* wild type/*erm*(41) M type] genotype groups. Patients infected with the resistant group isolates showed a significantly longer initial sputum medium conversion time: 12 months versus 7 months for the sensitive group (*P* = 0.004).

All patients enrolled in the study were treated with a standard combination of antibiotics based upon CLA. Patients infected with the CLA-sensitive genotype group isolates were significantly more likely to demonstrate initial sputum conversion (Fig. 1 and Table 4) (*P* = 0.011). Times to initial sputum conversion also differed significantly between the CLA-sensitive and CLA-resistant genotype groups (*P* = 004). Sputum relapse after initial conversion to negative occurred in both groups and did not differ significantly. The proportion of patients whose sputa converted and remained culture-negative during the follow-up period was significantly greater in the CLA-sensitive than in the CLA-resistant genotype group (61.3% versus 30.4%, respectively; *P* = 0.013). Radiographic improvement rates were significantly higher in patients infected with the CLA-sensitive genotype group isolates than in patients infected with the CLA-resistant genotype group (*P* = 0.006). The effective treatment response evaluated by radiology and microbiology was also significantly greater for the CLA-sensitive genotype group than for the CLA-resistant genotype group (*P* < 0.001).

**TABLE 4:** Treatment outcomes for CLA-resistant and -sensitive genotype groups

Parameter	Value^*a*^		*P* value
	Resistant group (*n* = 69)	Sensitive group (*n* = 31)	
Median duration of treatment [mo (IQR)]	18 (9–30)	15 (9–22)	0.260
Sputum result			0.013
Conversion to stable negative	21 (30.4)	19 (61.3)	
Failure to convert	39 (56.5)	9 (29.0)	
Relapse after conversion to negative	9 (13.0)	3 (9.7)	
Initial smear/culture conversion			
No. of patients who initially converted	30 (43.5)	22 (71.0)	0.011
Median time to initial conversion [mo (IQR)]	12 (6–23)	7 (5–11)	0.004
Radiological result			
Improved	25 (36.2)	22 (71.0)	0.006
No change	24 (34.8)	5 (16.1)	
Progressed	20 (30.0)	4 (12.9)	
Final treatment response			
Effective	30 (43.5)	26 (83.9)	<0.001
Failure	39 (56.5)	5 (16.1)	

a The data are the number and (percentage) of patients in each group unless otherwise indicated.

In multivariate analysis, the genotype was a reliable predictor of the response of M. abscessus lung disease to treatment (odds ratio [OR] = 0.185; 95% confidence interval [CI], 0.059 to 0.579; *P* = 0.004) (Table 5). All other characteristics, i.e., age, sex, body mass index (BMI), colony morphology, and CT imaging, were nonpredictors.

**TABLE 5:** Univariate and multivariate analyses of factors affecting combination antibiotic treatment

Variable	Unadjusted OR (95% CI)	*P* value	Adjusted OR (95% CI)	*P* value
Age (yr)				
>58	0.72 (0.33–1.60)	0.421		
<58	1			
Sex				
Male	1.93 (0.86–4.36)	0.113		
Female	1			
BMI				
>20.0	1.10 (0.48–2.35)	0.884		
<20.0	1			
Resistance of isolates				
Resistant	0.15 (0.051–0.431)	<0.001	0.185 (0.059–0.579)	0.004
Sensitive	1		1	
Initial morphotype				
Rough	0.86 (0.39–1.91)	0.708		
Smooth	1			
Positive AFB smear				
Yes	1.13 (0.50–2.56)	0.765		
No	1			
Bronchiectasis				
Yes	1.98 (0.32–12.37)	0.467		
No	1			
Tree-in-bud pattern				
Yes	2.91 (1.14–7.42)	0.025	2.217 (0.810–6.068)	0.121
No	1		1	
Cavity				
Yes	0.39 (0.17–0.90)	0.027	0.776 (0.298–2.022)	0.603
No	1		1	
Completed the initial 4 wk of treatment				
Yes	1.38 (0.55–3.45)	0.498		
No	1			

## DISCUSSION

The study reported here was the first undertaken to explore and correlate the differences in treatment outcomes of M. abscessus lung disease patients with the CLA susceptibility genotype of clinical isolates. We found that the treatment results for patients infected with isolates with the CLA-sensitive M. abscessus genotype were far superior to the results for patients infected with isolates with the CLA-resistant genotype evaluated in terms of sputum conversion rate, duration of initial sputum conversion, radiological improvement, and efficacy. Treatment outcome, however, was independent of all other factors examined, which included BMI, colony morphology, and radiological images.

In 2006, the M. abscessus complex was first divided into M. abscessus subsp. abscessus and M. abscessus subsp. massiliense based upon differences in the *rpoB* gene (24). In 2011, Bastian and coworkers reported that variations in the *erm*(41) genotype influenced the sensitivity of these subtypes to CLA *in vitro* (15). The clinical characteristics and treatment outcomes of patients infected with M. abscessus subsp. abscessus and M. abscessus subsp. massiliense differed in subsequent studies (17–20). Patients infected with M. abscessus subsp. massiliense usually responded better to treatment due, in part, to the CLA sensitivity of the organism. Several genotypes are associated with CLA sensitivity and -resistance: *rrl* mutant/wild type, *erm*(41)T28, *erm*(41)C28, and *erm*(41) M type. In the study described here, clinical isolates were grouped into these genotypes rather than M. abscessus subsp. abscessus and M. abscessus subsp. massiliense, and the responses of patients to standard, CLA-based treatment were assessed and compared. The response of the CLA-sensitive genotype group was significantly superior to that of the CLA-resistant genotype group judged in terms of the sputum conversion rate, radiological improvement, duration of initial sputum conversion results, and treatment efficacy. While lung disease patients infected with M. abscessus subsp. abscessus [*erm*(41)C28 genotype] isolates may exhibit a better response to combination CLA treatment, the response of patients infected with M. abscessus subsp. massiliense isolates expressing the 2058-2059 *rrl* mutation was often much worse. As such, CLA susceptibility genotyping is more accurate than subtyping as an approach to predicting the treatment outcomes of patients with M. abscessus lung disease (46.4% versus 42.9% true-positive rates, respectively).

The effect of BMI on the treatment outcomes of patients with NTM lung disease was demonstrated in several studies (25, 26). A recent retrospective study suggested that, in addition to CLA sensitivity, BMI was a factor that affected the success of M. abscessus lung disease treatment (20). This suggestion, however, was not confirmed by the present study. The overall BMIs of M. abscessus lung disease patients enrolled in our study were low; moreover, multivariate analysis failed to support its value in predicting an effective treatment outcome. This finding is consistent with results reported by other investigators (19). Similarly, predictions concerning the outcome of antibiotic therapy based upon symptoms or CT imaging are unrealistic. While hemoptysis and cavity-like manifestations were more common among the patients infected with CLA-resistant genotype M. abscessus, these factors failed to predict the prognosis upon multivariate analysis.

Patients infected with M. abscessus characterized by a rough-type colony exhibited a high incidence of cavities in CT images. This finding is consistent with the conclusion that rough-type strains usually exhibit higher virulence and pathogenicity. Unlike previous studies (19), however, we found that the initial colony morphology failed to correlate with the final radiologic improvement rate or treatment efficacy (Table 2). Jonsson and coworkers reported a significant increase in the number of rough colonies during the course of infection and the occurrence of smooth-to-rough colony conversion (27). Thus, we speculate that colony morphology is associated only with pathogenicity and the pathogenesis of infection and is not a reliable predictor of treatment efficacy.

The study described here has several limitations. First, only a relative small number of isolates exhibited the *rrl* mutation and *erm*(41)C28 genotypes; consequently, their characteristics may not be representative. Solidifying their characteristics will require the enrollment of more patients infected with isolates exhibiting the *rrl* mutant and *erm*(41)C28 genotypes in future studies. Second, a minority of patients relapsed following initially successful treatment (see Table S2 in the supplemental material). Conceivably, these relapses were due to subsequent infection by a different M. abscessus strain or genotype. In the absence of dynamic follow-up, our study failed to determine whether recurrence occurred due to reinfection by a different M. abscessus strain.

In conclusion, there was a significant difference in treatment outcomes for patients infected with CLA-resistant and -sensitive M. abscessus genotype isolates. The CLA-sensitive genotype group was significantly superior in sputum conversion rate, initial sputum conversion time, radiological improvement, and treatment efficacy. Accurate genotyping is an important factor in predicting the efficacy of combination therapy with CLA-based antibiotics. Rapid genotyping should help clinicians optimize therapeutic strategies, especially in cases of critically ill patients who cannot wait weeks for culture and susceptibility testing. Genotyping would also be effective as a diagnostic approach in areas where facilities for mycobacterial culture and susceptibility testing are unavailable.

## MATERIALS AND METHODS

### Study population.

**FIG 2:**
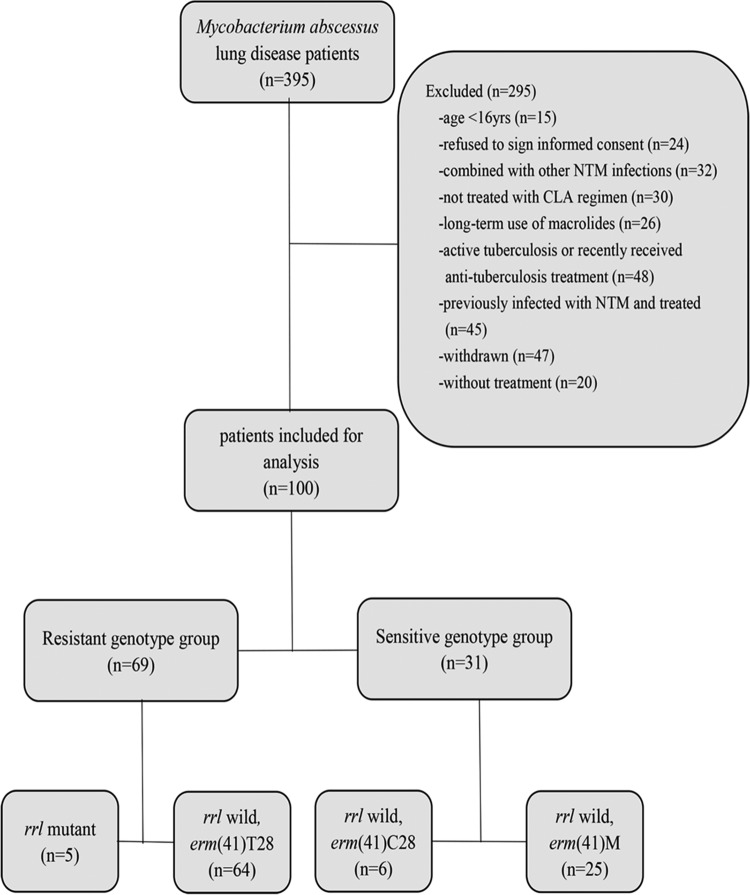
Flow diagram of the study. One hundred M. abscessus lung disease patients who conformed to the inclusion criteria were enrolled. Sixty-nine patients were in the CLA-resistant genotype group, including 5 patients infected with *rrl* 2058-2059 mutants and 64 *erm*(41)T28-type-infected patients; 31 belonged to the CLA-sensitive group, including 6 *erm*(41)C28- and 25 *erm*(41) M-type-infected patients.

A retrospective review of the medical records of all patients with M. abscessus lung disease was conducted between January 2012 and December 2015 at the Shanghai Pulmonary Hospital. Patient inclusion criteria were as follows: (i) age, >16 years; (ii) underwent initial diagnosis and treatment at the Shanghai Pulmonary Hospital in accordance with the 2007 American Thoracic Society/Infectious Disease Society of America (ATS/IDSA) guidelines; (iii) received oral CLA-based combination treatment; (iv) follow-up period lasted more than 6 months. The exclusion criteria were as follows: (i) age, <16 years; (ii) history of NTM lung disease; (iii) lack of critical visit data (e.g., regular sputum culture or CT examination), failure to follow up, or death from non-M. abscessus lung disease-related causes; (iv) treatment did not include oral CLA; (v) history of long-term macrolide drug treatment; (vi) diagnosed with active tuberculosis or received antituberculosis treatment within 3 months prior to study enrollment; (vii) coinfected with another nontuberculosis mycobacterium; (viii) refused to sign informed consent form; (ix) AIDS. In addition, patients with cystic fibrosis were not included in the study; notably, cystic fibrosis is extremely rare among Asian patients. A detailed, patient enrollment flow chart is shown in Fig. 2. This study was approved by the Ethics Committees of Shanghai Pulmonary Hospital and Tongji University School of Medicine, ethics number K17-150. All participants signed informed consent forms before enrollment.

### Collection, identification, and preservation of bacteria.

All the clinical M. abscessus isolates used in this study were preserved in the Clinical Microbiology Laboratory of Shanghai Pulmonary Hospital. Shanghai Pulmonary Hospital is one of the designated treatment centers for tuberculosis and NTM disease in China, attracting NTM disease cases nationwide. M. abscessus isolates were obtained from sputum and bronchoalveolar lavage fluid. Samples were transferred to Lowenstein-Jensen (L-J) agar plates after treatment with 4% NaOH. Smears prepared from the bacterial colonies that grew were stained and examined microscopically to identify the acid-fast organisms. To select further NTM, positive colonies were inoculated and cultured in L-J medium containing 0.5 mg/ml P-nitrobenzoic acid and 5 mg/ml 2-thiophenecarboxylic acid hydrazide for 1 to 2 weeks at 37°C. Bacterial isolates that grew rapidly were selected for molecular typing by PCR. The bacteria were digested with 1 mg/ml lysozyme and 1 mg/ml proteinase K, and the DNA was extracted with phenol-chloroform. First, the *rpoB* gene was amplified by PCR, and the DNA sequences were determined. To confirm the M. abscessus complexes, 754 bp of the DNA segment was subjected to BLAST analysis. Second, the *erm*(41) gene was amplified, and the DNA sequence was analyzed to identify and differentiate M. abscessus subsp. massiliense, M. abscessus subsp. abscessus, and M. abscessus subsp. bolletii. Finally, the *PRA-hsp65* gene was compared to an online reference (http://app.chuv.ch/prasite/index.html) to confirm the M. abscessus subsp. abscessus and M. abscessus subsp. bolletii identifications. M. abscessus subsp. *bolletii* was excluded from the study because it is essentially absent in China. Identified M. abscessus subsp. abscessus and M. abscessus subsp. massiliense isolates, stored at −80°C, were subsequently recovered for microbiology and molecular biology studies.

### Identification of colony morphology.

Single colonies were obtained from frozen M. abscessus isolates by growth on Middlebrook 7H10 agar plates supplemented with 10% oleic acid-albumin-dextrose-catalase. The colonies were classified macroscopically as smooth or rough. If isolates gave rise to colonies of both morphotypes, a colony of each type was analyzed separately, and the identity was established by whole-genome sequencing.

### Genotype analysis.

Genomic information for all isolates was obtained by whole-genome sequencing. Single nucleotide polymorphism (SNP) analysis was performed using the NCBI GenBank database and BLAST algorithm. The following genotypes were of specific interest: *erm*(41) [including *erm*(41)C28, *erm*(41)T28, and *erm*(41) M type], *rll* wild type, and *rrl* 2058-2059 mutant.

### (i) Whole-genome sequencing.

Detailed methods were published previously by us (28). DNA was extracted according to the method of Somerville and coworkers (29), and paired-end libraries with insert sizes of ∼400 bp were prepared following Illumina's standard genomic DNA library preparation protocol (Illumina, San Diego, CA, USA). After shearing, ligating, and PCR, the qualified Illumina paired-end library was used for Illumina HiSeq sequencing (paired-end 150 bp × 2). The default parameters of the SPAdes software (version v.3.6.0) (http://bioinf.spbau.ru/en/spades) were used to assemble the genome draft (30). The assembled product was evaluated using QUAST (version v.2.3) (31; http://quast.bioinf.spbau.ru/).

### (ii) SNP analysis.

The NCBI Nucleotide BLAST program was used for SNP analysis. The standard ATCC 19977 (NC_010397.1) M. abscessus strain served as the reference for *rrl* and *erm*(41)T28, CR5701 (HQ127366.1) was used as the reference strain for *erm*(41)C28, and CCUG48898 (AP014547.1) was the reference for M type.

### Drug sensitivity assay.

Antibiotic sensitivity was determined by the microdilution method. Sulfonamides, moxifloxacin, cefoxitin, amikacin, doxycycline, tigecycline, CLA, linezolid, imipenem, and tobramycin are among the most common antibiotics used to treat M. abscessus infections; each was tested (TREK Diagnostic Systems, Brooklyn Heights, OH, USA). CLA resistance was assessed at 3 days and 14 days after M. abscessus exposure. Antibiotics' susceptible and resistant breakpoints were interpreted according to Clinical and Laboratory Standards Institute (CLSI) document M24-A2. Staphylococcus aureus (ATCC 29213; American Type Culture Collection, Manassas, VA, USA) served as the control reference strain.

### Treatment regimen and efficacy evaluation.

All patients were treated with antibiotics as follows: an initial 4-week course of amikacin (15 mg/kg of body weight/day in two equal doses) combined with cefoxitin (200 mg/kg/day with a maximum of 12 g/day in three equal doses) by intravenous administration. CLA was also administered orally from the beginning of therapy. After 4 weeks, an oral regimen of CLA combined with levofloxacin or moxifloxacin was given. If an adverse reaction to either amikacin or cefoxitin occurred, the regimen was replaced with imipenem (500 mg three times a day), linezolid (600 mg once every 12 h), or tigecycline (100 mg initially, followed by 50 mg every 12 h). CLA was administered continually throughout the course of treatment as recommended in the guidelines.

All the patients underwent chest CT examination, as well as sputum smears and culture, regularly. Therapeutic efficacy was determined according to the results of microbiological examination and radiological changes. The clinical characteristics, sputum culture conversion rate and time, radiological improvement rate, and microbiological characteristics of each genotype group were compared. Culture conversion was defined as three consecutive negative cultures from sputum specimens. Effective treatment was defined as sputum culture negative or significant pulmonary lesion resolution without recurrence during the observation period. Ineffective treatment included failure to achieve culture and smear conversion, recurrence after initial culture conversion, and appearance of increased or stable lesions in CT scans.

### Statistical analysis.

All statistical analyses were conducted using SPSS20.0 (IBM, Armonk, NY, USA). The data were compared using Student's *t* test or the Mann-Whitney *U* test for continuous variables and the Pearson χ^2^ test or Fisher exact test for categorical variables. *P* values of <0.05 were considered statistically significant in a 2-tailed analysis. Times to initial culture conversion were compared using the Kaplan-Meier method. Potential predictors of the treatment response were assessed by multivariable logistic regression. In the logistic regression models, variables with *P* values of <0.1 in the univariable analysis were included in the multivariable analysis.

### Accession number(s).

The accession numbers for all the M. abscessus isolates sequenced in this study are available at DDBJ/ENA/GenBank under BioProject PRJNA398137.

## Supplementary Material

Supplemental material
