# Supplementary material for: The Clarithromycin Susceptibility Genotype Affects the Treatment Outcome of Patients with Mycobacterium abscessus Lung Disease
Source: Antimicrob Agents Chemother. 2018 Apr 26;62(5):e02360-17. doi: 10.1128/AAC.02360-17 (PMC5923093; doi:10.1128/AAC.02360-17)
Supplement: Supplemental material [file supp_62_5_e02360-17__index.html]

Supplemental material 

# The Clarithromycin Susceptibility Genotype Affects the Treatment Outcome of Patients with Mycobacterium abscessus Lung Disease

## Supplemental material

- Supplemental file 1 -

  Tables S1 and S2 and Fig. S1

  PDF, 204K
