# Supplementary material for: The Clarithromycin Susceptibility Genotype Affects the Treatment Outcome of Patients with Mycobacterium abscessus Lung Disease
Source: Antimicrob Agents Chemother. 2018 Apr 26;62(5):e02360-17. doi: 10.1128/AAC.02360-17 (PMC5923093; doi:10.1128/AAC.02360-17)
Supplement: Supplemental material [file AAC.02360-17_zac005187122s1.pdf]

Supplementary Table 1. Antibiotic resistance of all *M. abscessus* isolates[a]

Antibiotic	MIC (mg/mL)			Isolates n (%)			
				Resistant genotype (n=69)		Sensitive genotype (n=31)	
	Sensitive	Intermediate	Resistant	*rrl* mut (n=5)	*rrl* wild, *erm*(41)T28 (n=64)	*rrl* wild, *erm*(41)C28 (n=6)	*rrl* wild, *erm*(41) M (n=25)
CLA (resistant before induction)	<2	4	>8	5 (100.0)	27 (42.2)	0 (0.0)	0 (0.0)
CLA (resistant after induction)	<2	4	>8	5 (100.0)	63 (98.4)	0 (0.0)	1 (28.0)
AMI (amikacin)	<16	32	>64	0 (0)	1 (1.6)	1 (16.7)	0 (0)
SXT (sulfonamides)	<2	nrb[b]	>4	2 (40.0)	36 (56.3)	3 (50.0)	15 (60.0)
MXF (moxifloxacin)	<1	2	>4	5 (100.0)	63 (98.4)	6 (100)	24 (96.0)
FOX (cefoxitin)	<16	32-64	>128	1 (20.0)	42 (65.6)	5 (83.3)	17 (68.0)
DOX (doxycycline)	<1	2-4	>8	5 (100.0)	63 (98.4)	6 (100)	25 (100.0)
TGC (tigecycline)	<1	nrb	nrb	nd[c]	nd	nd	nd
LZD (linezolid)	<8	16	>32	3 (60.0)	30 (46.9)	3 (50.0)	10 (40.0)
IMI (imipenem)	<4	8-16	>32	5 (100.0)	61 (95.3)	6 (100)	24 (96.0)
TOB (tobramycin)	<2	4	>8	4 (80.0)	56 (87.5)	6 (100)	24 (96.0)

[a]The *erm*(41) sequevar-dependent resistance of 100 *M. abscessus* isolates to the antibiotic indicated was determined by the microdilution method. The incubation time was 3 days (before induction) and 14 days (after induction) for CLA, and 3 days for the other antibiotics listed. MIC = minimum inhibitory concentration.
[b]no recommended breakpoint.
[c]no data.

Supplementary Table 2. Relapse among patients infected with four *M. abscessus* genotype groups

	Resistant group		Sensitive group		*P* value
	rrl (n=5)	T28 (n=64)	C28 (n=6)	M (n=25)	
Duration of treatment, mean month	10.5	8.1	6.0	1.0	0.257
Patients defined as relapse*	2 (40.0)	8 (12.5)	3 (50.0)	1 (4.0)	0.009
Sputum relapse after initial smear/culture conversion	2 (40.0)	7 (10.9)	2 (33.3)	1 (4.0)	0.049
Radiological progressed without sputum relapse	2 (40.0)	8 (12.5)	3 (50.0)	0 (0)	0.003

*Data are the number and (percentage) of patients infected with each genotype. Significant differences between the resistant and sensitive groups are indicated by a *P* value <0.05.

Supplementary Figure 1. Comparison of initial sputum smear/culture conversion rate among 4 genotypes

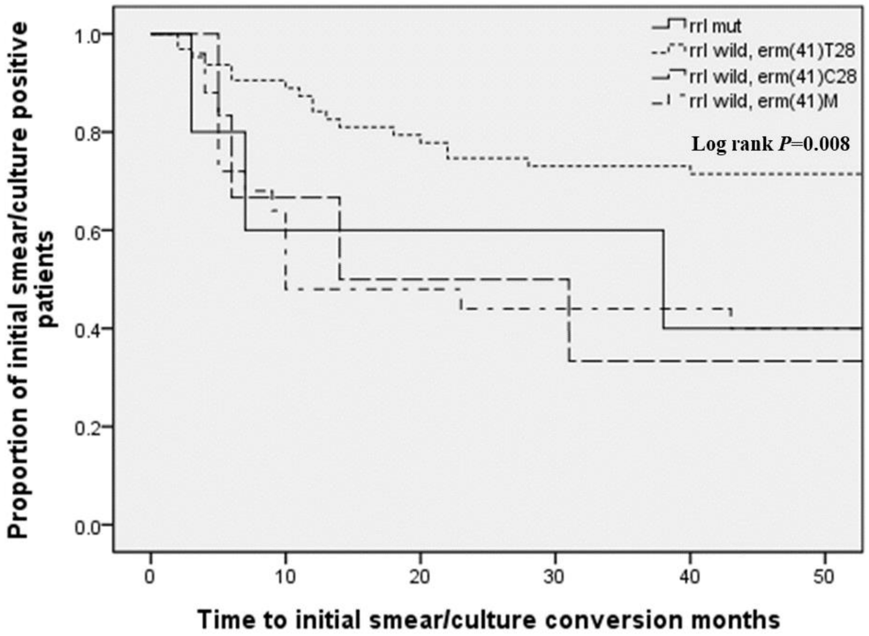

Supplementary Figure 1. Comparison of initial sputum smear/culture conversion rate among patients infected with the genotype groups indicated. Patients infected with the resistant, *rrl* mut and *rrl* wild/*erm*(41)T28 genotypes showed a significantly longer initial sputum medium conversion time than patients infected with the two sensitive genotypes, *P* = 0.008.
